# The role of pregnancy intendedness and prenatal contraceptive counseling on postpartum contraceptive use

**DOI:** 10.1186/s40834-020-00127-4

**Published:** 2020-10-22

**Authors:** Karina M. Shreffler, Stacy Tiemeyer, Jameca R. Price, Lance T. Frye

**Affiliations:** 1 Oklahoma State University, 700 N. Greenwood Ave, Tulsa, OK 74106 USA; 2 University of Oklahoma Health Sciences Center, 4502 E. 41st St, Tulsa, OK 74135 USA; 3 Oklahoma State University Center for Health Sciences, 1111 W. 17th St, Tulsa, OK 74107 USA

**Keywords:** Family planning, Contraception, Counseling, LARC, Unintended pregnancy, Ambivalence

## Abstract

**Background:**

The study was conducted to prospectively examine how pregnancy intendedness and prenatal provider counseling about postpartum contraceptive options are associated with lack of contraception use at 6 months post-birth (e.g., increased risk for a short interpregnancy interval).

**Methods:**

Logistic regression models were used to examine risk for no postpartum contraception use among a sample of low-income and racially/ethnically diverse women recruited from two metropolitan perinatal clinics in Tulsa, OK.

**Results:**

Women who reported that they were trying to get pregnant or “okay either way” about getting pregnant had significantly lower odds of using contraception at 6 months post childbirth than those who had unintended pregnancies. Having providers who discussed postpartum contraceptive options during pregnancy significantly increased the odds of contraceptive uptake among those who were planning or ambivalent about their pregnancies.

**Conclusions:**

Intentions of a current pregnancy and provider contraceptive counseling matter for postpartum contraceptive use and the associated risk for a short interval subsequent pregnancy. Provider contraceptive counseling that accounts for the intendedness of a current pregnancy may offer a more targeted approach to prevent a short interval subsequent pregnancy.

## Background

Nearly half (45%) of the 6.1 million pregnancies in the US each year are unintended [1]. The public health impact of unintended pregnancy is considerable: women who carry an unintended pregnancy to term are more likely to delay prenatal care, use alcohol and tobacco, and experience low infant birth weight, preterm birth, and maternal morbidity and mortality [2–6]. Most unintended pregnancies in the US occur because women and their partners do not use contraception consistently and effectively [7].

The most effective form of female contraception is tubal ligation [8]. Though so-called “short-term” contraceptive methods (e.g., oral and barrier methods of contraception) tend to be quite effective if used correctly [9], long-acting reversible contraception (LARC)--such an intra-uterine device (IUD) or implant—are highly effective at preventing first-time [10], rapid repeat [11], and unintended pregnancy [12]. ACOG and the American Academy of Pediatrics (AAP) recommend LARC methods as the best option for adolescents and women [13, 14]. Despite the proven effectiveness of LARC methods, their use in 2013 was not as widespread as condoms (32%) or birth control pills (27%) [12]. Approximately 14% of reproductive-aged women (ages 15–44) used an IUD or implant in 2013, though that is nearly a five-fold increase since 2002 [12, 15].

Promoting postpartum contraception such as LARC insertion has been identified as an effective and timely strategy to reduce inadequate birth spacing and unintended pregnancy [16]. Lack of knowledge about contraception, both perceived and actual, has been identified as the primary barrier to contraceptive uptake [17]. Women who discussed LARC methods with their providers are more than 13 times more likely to adopt intrauterine contraception than those who did not discuss the method with their providers [18]. Prenatal facilities differ considerably on practices regarding postpartum LARC insertion and communication with patients [19], but even within the same prenatal clinic, uptake of postpartum contraception can differ by patient characteristics. For example, women who have public (e.g., Medicaid) insurance as compared to private insurance are more likely to receive postpartum LARC insertion [20]. Yet it is unclear how characteristics of a prior pregnancy predict postpartum contraceptive use. Identifying circumstances that predict or disrupt postpartum uptake of effective contraception is therefore a critical need. Prior qualitative research with a non-pregnant sample of reproductive-age women found that ambivalence about getting pregnant is associated with being less receptive to LARC methods [21]. In the current prospective study of a sample of pregnant women ages 15–39, we examine how the intendedness of a pregnancy predicts risk for a subsequent pregnancy at 6 months postpartum as measured by contraceptive non-use and whether provider recommendations moderate the impact of intendedness.

## Methods

### Sample

We conducted enrollment for the longitudinal clinic-based cohort study between October 2016 and May 2017. After securing Institutional Review Board approval at the participating authors’ institution, pregnant women were recruited from the two university-affiliated perinatal clinics in a city in the South-Central U.S. The participating clinics were purposively selected because they serve racially diverse, but socioeconomically disadvantaged and medically underserved patient populations. Recruitment of study participants took place in the obstetric practices of the participating centers during prenatal visits. Research and clinical partners worked together to develop a screening, recruitment and participant transfer protocol. All pregnant patients seen by providers during the designated recruitment times were screened for study eligibility. A screen to determine eligibility was completed by nursing staff and provided to the research staff. A patient was eligible for participation if she was 15+ years old and less than 28 weeks pregnant. The sample for this paper comes from the 177 patients who agreed to participate. The sample size at recruitment was determined by a power calculation for a study of rapid repeat pregnancy.

### Patient and public involvement

Neither patients nor the public were involved in the design, conduct, reporting, or dissemination plans of the research study.

### Measures

The measures used in this study come from longitudinal data collected during pregnancy and within the first 6 months postpartum. Most women completed the first survey during their first trimester (72%), and 90% of the women were less than 20 weeks pregnant. The first survey asked demographic, medical and pregnancy history, as well as a number of psychosocial measures. Additional survey assessments were sent to participants via text and email during their second and third trimesters, 2 months postpartum, 6 months postpartum, and 1 year postpartum.

#### Dependent variable

Our outcome variable, *contraceptive use* was assessed using post-birth survey data. From the six-month post-birth survey, we created a dichotomous variable indicating contraceptive use; respondents who indicated that they were not consistently using any form of contraception despite having regular heterosexual intercourse were coded as 0, with respondents either using contraception or not having sexual intercourse coded as 1. For descriptive purposes, we also created dummy variables for methods of contraception, though small cell sizes within the different methods prevented multivariate analysis.

#### Independent variables

To measure *pregnancy intendedness*, we used a question from the National Survey of Fertility Barriers that asked women about their reproductive behaviors at the time of their pregnancy: “Right before you got pregnant, would you say you were trying to get pregnant, trying NOT to get pregnant, or okay either way?” Responses were coded as dummy variables for “intended” (e.g., trying to get pregnant) and “ambivalent” (e.g., okay either way), as compared to those who were “unintended” (e.g., avoiding pregnancy) as the reference group.

We measured *contraceptive counseling* at the two-week post-birth survey with a question asking women, “Did your doctor, or any healthcare provider, discuss long-acting birth control options with you?” Response options were coded as yes (=1), no (=0), and don’t know (=0).

#### Control variables

Our controls for the following sociodemographic variables collected at the first assessment include no desire for more children, race/ethnicity, parity, age, relationship status, economic hardship, and clinic where recruitment occurred. *No desire for more children* was measured with a question asking women if they wanted more children at any point after giving birth; no was coded as 1 with yes or unsure coded as 0. *Race/Ethnicity* was measured using Census coding recommendations with four categories: non-Hispanic white, non-Hispanic black, Hispanic, and Native American. *Parity* was calculated from reported live births as a continuous variable. *Age* was measured in years. We included a dummy variable indicating the respondent was in a *union* if she was married or cohabitating with a partner. *Economic hardship* was measured with seven items and included, “In the past year, did any of the following happen to you or members of your household because of a shortage of money” …. “went without meals;” “could not pay the mortgage or rent on time;” and “asked for financial help from friends or family.” “Yes” responses were coded as 1 and summed to create an index of economic hardship. Additionally, although both medical practices served disadvantaged populations, there were a few differences between the obstetric practices. First, one practice served a slightly more urban population, and we observed differences in racial/ethnic distributions. Second, there were differences in institutional structures and affiliations. *Clinic 1* has the ability to do LARC placement at the time of delivery, whereas the other practice is affiliated with a religious-based institution that does not allow placement of LARC at delivery, though they can offer it at the post-birth clinic visit. Both clinics offer LARC at no charge to patients on public insurance. Due to these differences, we controlled for the location participants were recruited from in our analysis. Finally, we also controlled for whether the participants reported *prior unintended pregnancies* (1 = yes; 0 = no).

### Analytic plan

We conducted logistic regression analyses to examine how pregnancy intendedness and provider contraceptive counseling predicted contraceptive non-use at 6 months. We ran all analyses in Stata. All variables in our analysis had fewer than 5% missing values. We used Stata’s mi package to impute missing values on variables using chained equations in Stata to fit five imputation models.

## Results

### Sample characteristics

**Fig. 1:**
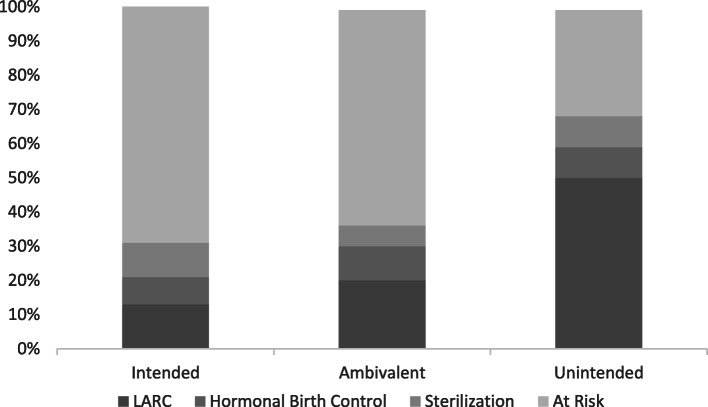
Contraception Use at 6 Months Postpartum by Pregnancy Intendedness

The analytical sample for the study included the approximately 71% of respondents who completed the survey assessments during pregnancy through the six-month post-birth follow up. This resulted in a final sample of 125 participants. Table 1 shows the descriptive statistics for the full sample as well as split into pregnancy intendedness groups. There was a fairly even split between pregnancy intendedness, with the largest group (40%) of the sample reporting that they were ambivalent about getting pregnant, 34% reporting that their pregnancies were intended, and 26% reporting that they were avoiding pregnancy (e.g., unintended) when it occurred. Two-thirds (66%) of participants reported that their providers discussed post-birth contraceptive options during pregnancy with them. The majority of the sample (56%) were at risk for a subsequent pregnancy at 6 months post-birth, meaning that they were sexually active and not using any form of contraception. The “unintended” group was the only one in which the majority of the women (69%) were using contraception 6 months after giving birth. Post-birth contraceptive methods by pregnancy intendedness status are depicted visually in Fig. 1. LARC methods were most popular among those with an unintended pregnancy, and among those who were ambivalent about getting pregnant, LARC was the most common contraceptive method used among those using contraception. More than half of those who had intended or were ambivalent about their pregnancies reported that they were sexually active and not using contraception 6 months after giving birth.

**Table 1:** Contraceptive Use and Contraceptive Counseling by Pregnancy Intention Status

Characteristics	Total (*n* = 125)	Intended	Ambivalent (*n* = 50)	Unintended (*n* = 33)
		(*n* = 42)		
	%/M (SD)	%/M (SD)	%/M (SD)	%/M (SD)
Contraceptive use at 6 months postpartum	44%	32%	38%	69%
Contraceptive counseling during pregnancy	66%	55%	76%	66%
Control variables				
Desires no more births	37%	24%	40%	50%
Race/Ethnicity				
White	41%	46%	44%	28%
Black	28%	27%	22%	41%
Hispanic	14%	15%	12%	16%
Native American	17%	12%	22%	16%
Parity	1.27 (1.31)	1.29 (1.45)	1.08 (1.24)	1.56 (1.21)
Age (years)	25.84 (5.47)	26.73 (5.82)	25.86 (5.76)	24.66 (4.87)
Residential partner	65%	76%	71%	41%
Economic hardship	1.56 (1.82)	1.71 (1.92)	1.55 (1.91)	1.34 (1.92)
Clinic 1	67%	66%	66%	72%
Ever had unintended pregnancy	57%	48%	55%	70%

The logistic regression results are presented in Table 2. With all control variables included in the analysis (Model 1), women who reported their pregnancies were intended and those who were ambivalent about getting pregnant had significantly lower odds of using contraception at six-months post birth than those who reported unintended pregnancies (OR = .19, *p* < .01 and OR = .30, *p* < 05, respectively). Latina and Native American women had lower odds of using contraception at 6 months postpartum than white women (OR = .23, *p* < .05; OR = .19, *p* < .05, respectively).

**Table 2:** Logistic Regression Analysis Predicting Contraceptive Use at 6 Months Postpartum, with 95% CI (*N* = 125)

	M1		M2	
	aOR^a^	*p*-value	aOR^a^	*p*-value
Pregnancy intendedness				
Unintended (reference)				
Intended	.19 [.06,.65]	<.01	.03 [.00,.33]	<.01
Ambivalent	.30 [.10,.88]	<.05	.02 [.00,.35]	<.01
LARC counseling during pregnancy	.99 [.38,2.62]		.10 [.01,1.09]	
Control variables				
Desires no more children	1.44 [.52,3.98]		1.50 [.52,4.31]	
Race				
White (reference)				
Black	.47 [.15,1.48]		.39 [.12,1.26]	
Hispanic	.23 [.05,.1.00]	<.05	.27 [.06,1.18]	
Native American	.19 [.05,.71]	<.05	.18 [.04,.77]	<.05
Parity	1.82 [1.09,3.04]	<.05	1.79 [1.06,3.01]	<.05
Age (years)	.91 [.82,1.02]		.91 [.82,1.02]	
Residential partner	.79 [.31,2.05]		.75 [.27,2.07]	
Economic hardship	1.02 [.81,1.28]		1.03 [.81,1.31]	
Clinic 1	2.36 [.80,7.01]		2.40 [.75,7.63]	
Ever had unintended pregnancy	.48 [.15,1.51]		.52 [.17,1.75]	
Pregnancy intention X contraceptive counseling				
Intended X Counseling			15.87 [.98,255.87]	
Ambivalent X Counseling			32.53 [1.56,586.32]	<.05

^a^ *aOR* Adjusted odds ratio

**Fig. 2:**
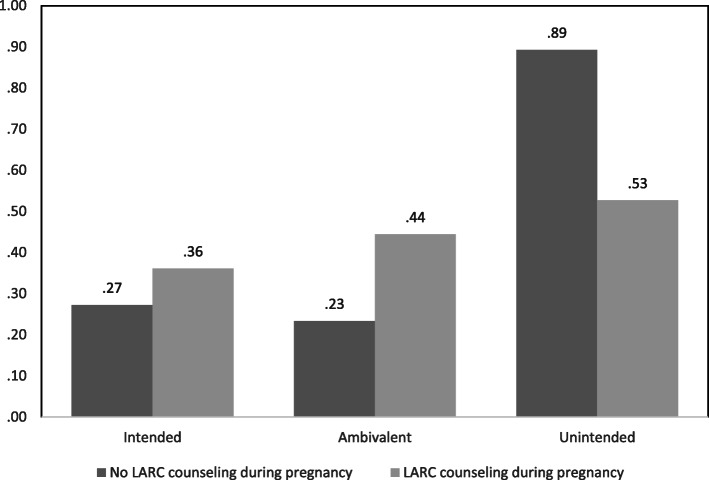
Predicted Probabilities of Contraceptive Use at 6 Months Postpartum by Pregnancy Intendedness

**Fig. 3:**
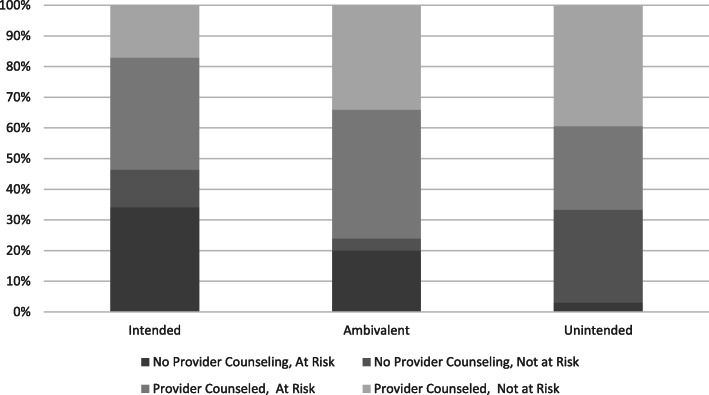
Provider Counseling and Risk for Short Interpregnancy Interval at 6 Months Postpartum by Pregnancy Intendedness

In Model 2, interactions between pregnancy intendedness and provider contraceptive counseling were added to the analysis. Findings indicate greater odds of contraceptive use among those who were ambivalent about their pregnancies when providers counseled them about post-.

birth contraception options during their pregnancy. To ease readability of the interaction findings, Fig. 2 presents the predicted probabilities of contraceptive use by pregnancy intention and provider counseling. Results indicate that among those who were ambivalent about their pregnancies, lack of provider contraceptive counseling was associated with significantly lower odds of contraceptive use at 6 months postpartum despite being sexually active. The findings for those with unintended pregnancies were not in the expected direction, so we also created Fig. 3 to better understand how pregnancy intentions and provider counseling were associated with pregnancy risk at 6 months postpartum. Results suggest that those who were ambivalent about their pregnancies received the most counseling, and that women with unintended pregnancies were more likely to opt for postpartum contraception, even without discussions with their provider about post-birth contraception options.

## Discussion

Using a prospective, clinic-based sample of low-income pregnant women, we examined whether pregnancy intendedness and provider postpartum contraceptive counseling predicted risk for contraceptive non-use 6 months after giving birth. The findings from this study were consistent with prior findings that women interested in avoiding a subsequent pregnancy are most likely to opt for postpartum contraception [22]. Our findings highlighted that women who reported their pregnancy was intended or that they were ambivalent about getting pregnant were significantly less likely to use contraception following birth. The significant moderation between pregnancy intendedness and provider contraceptive counseling revealed that among pregnant women who were ambivalent about getting pregnant, receiving counseling about contraceptive options available after birth significantly increased the odds of using contraception at 6 months post birth.

This finding has critical implications for practitioners who provide contraceptive counseling. Ambivalence about getting pregnant is common; women often perceive both positive and negative consequences if they become pregnant [23], and those who report ambivalence tend to overestimate the risks associated with more effective contraceptive methods (e.g., LARC methods) and overestimate the effectiveness of oral contraception [24]. Providers who identify their patients as ambivalent have the opportunity to ask their patients about perceived benefits and adverse consequences if they become pregnant and to inform their patients about different contraceptive methods and their effectiveness. Our results suggest that this is a particularly receptive group for contraceptive counseling that can reduce the risk of a short inter-pregnancy interval.

This analysis has several limitations. Due to the nature of the sample being recruited from clinics serving primarily patients receiving public healthcare coverage, we were unable to examine insurance differences in post-birth contraceptive coverage. All participants had the option for free LARC insertion post-birth, however, which may explain why economic hardship did not significantly predict post-birth contraceptive use. Additionally, due to the small sample size, we were not able to empirically examine different methods of contraception. Future research should include a larger sample to allow for analysis of a wider array of methods to gain insight into those choices and consequences for short interpregnancy intervals. Still, a substantial proportion of our participants (56%) are at risk for a subsequent pregnancy (e.g., not using any form of contraception despite engaging in regular, heterosexual intercourse) only 6 months following a birth. Nonetheless, these limitations are offset by the strengths of this analysis, which include the prospective nature of the study and a predominately low-income and diverse sample. Future research is needed to further explore the findings about pregnancy intendedness and provider contraceptive counseling. For example, were women with intended pregnancies less likely to receive provider counseling because they were worried that a long-acting method may prevent them from having more children? Did women with unintended pregnancies report that they did not discuss contraception with providers because they had already decided to pursue contraception following birth, thus making contraceptive counseling unnecessary for those highly motivated to seek it?

## Conclusion

Our study highlighted the role that healthcare providers can play in encouraging women to uptake highly effective forms of contraception to delay a subsequent birth—particularly among women who expressed ambivalence about pregnancy. In conversations with pregnant women about post-birth contraception, providers should consider asking women about the intendedness of their current pregnancy. Women who are highly motivated to avoid pregnancy are most likely to opt for LARC or another contraceptive method after a birth, but those who are interested in giving birth again in the future should be informed about the risks for short interpregnancy intervals and the effectiveness of different forms of contraception.

## Data Availability

All data are on a secure database accessible by the authors. The dataset analyzed during the current study is available from the corresponding author on reasonable request.

## Abbreviation

LARC: Long-acting reversible contraception
